# High-Intensity Exercise Training Impact on Cardiorespiratory Fitness, Gait Ability, and Balance in Stroke Survivors: A Systematic Review and Meta-Analysis

**DOI:** 10.3390/jcm13185498

**Published:** 2024-09-17

**Authors:** Alessio Baricich, Margherita Beatrice Borg, Marco Battaglia, Salvatore Facciorusso, Stefania Spina, Marco Invernizzi, Lorenza Scotti, Lucia Cosenza, Alessandro Picelli, Andrea Santamato

**Affiliations:** 1 Department of Biomedical Sciences, Humanitas University, Pieve Emanuele, 20072 Milan, Italy; alessio.baricich@hunimed.eu; 2 Rehabilitation Unit, IRCSS Humanitas Research Hospital, 20089 Milan, Italy; 3 Department of Health Sciences, Università del Piemonte Orientale “Amedeo Avogadro”, 28100 Novara, Italy; marco.battaglia@uniupo.it (M.B.); marco.invernizzi@med.uniupo.it (M.I.); 4 Physical Medicine and Rehabilitation Unit, AOU Maggiore della Carità University Hospital, 28100 Novara, Italy; lucia.cosenza@maggioreosp.novara.it; 5 Department of Medical and Surgical Sciences, Spasticity and Movement Disorders “ReSTaRt” Unit, Physical Medicine and Rehabilitation Section, University of Foggia, 71122 Foggia, Italy; s.facciorusso89@gmail.com (S.F.); spinastefania.ss@gmail.com (S.S.); andrea.santamato@unifg.it (A.S.); 6 Translational Medicine, Dipartimento Attività Integrate Ricerca e Innovazione (DAIRI), Azienda Ospedaliera Santi Antonio e Biagio e Cesare Arrigo, 15122 Alessandria, Italy; 7 Department of Translational Medicine, Università del Piemonte Orientale “Amedeo Avogadro”, 28100 Novara, Italy; lorenza.scotti@uniupo.it; 8 Department of Neurosciences, Biomedicine and Movement Sciences, University of Verona, 37129 Verona, Italy; alessandro.picelli@univr.it

**Keywords:** high-intensity exercise, high-intensity training, cardio-respiratory fitness, stroke, meta-analysis, rehabilitation

## Abstract

Stroke survivors commonly face challenges such as reduced physical activity and cardiorespiratory fitness (CRF) as well as balance and gait impairments, exacerbating their disability. While high-intensity exercise interventions have demonstrated some potential, their effects on these items remain uncertain. Therefore, our study aimed to investigate the impact of high-intensity training protocols on CRF, gait ability, and balance in stroke survivor populations. Two independent investigators systematically searched five databases for relevant RCTs following the PICO model. Through a systematic review of 25 RCTs published up to 31 May 2023, including adult first-stroke survivors, comparing high-intensity exercise training versus low-to-mild or no exercises, we evaluated outcomes such as the Six-Minute Walking Test (6 MWT), peak oxygen uptake (VO2peak), Ten-Meter Walk Test (10 MWT), Berg Balance Scale (BBS), and Timed Up and Go test (TUG). The protocol was registered in PROSPERO (registration number CRD42023456773). Meta-analyses indicated significant enhancements in CRF, as measured by 6 MWT and VO2peak, following high-intensity exercise interventions. However, no significant differences were observed in BBS, 10 MWT, and TUG. Our findings underscore the potential of high-intensity exercise interventions in ameliorating CRF among stroke survivors, although further research involving standardized protocols and long-term follow-ups is imperative to optimize rehabilitation outcomes.

## 1. Introduction

A stroke is an abrupt disruption of blood flow to the brain, leading to a decline in brain function. This interruption can be the result of either a blockage in an artery that supplies blood to the brain, commonly referred to as an ischemic stroke, or a consequence of a blood vessel rupture within the brain, referred to as hemorrhagic stroke [1]. Stroke is the foremost cause of disability globally and ranks as the second leading cause of death. The Global Stroke Factsheet published in 2022 reports that, from 1990 to 2019, stroke incidence increased by 70%, stroke-related deaths rose by 43%, stroke prevalence grew by 102%, and disability-adjusted life years (DALYs) attributed to stroke surged by 143% [2].

Stroke can lead to significant disabling consequences, particularly in terms of physical inactivity and deconditioning. The aftermath of a stroke often results in motor impairments, weakness, altered coordination, fatigue, and cognitive impairments contributing to reduced physical fitness, gait alterations, and imbalance. All these implications limit an individual’s ability to engage in physical activities. Prolonged periods of immobility and reduced physical activity contribute to muscle atrophy, and cardiovascular deconditioning, exacerbating the overall functional decline. Moreover, physical inactivity is not only a consequence of stroke but also a contributing factor to a vicious cycle of reduced fitness [3,4,5].

Cardiorespiratory fitness (CRF) is the capacity of the cardiovascular and respiratory systems to deliver oxygen to the muscles during prolonged physical activity. It is often measured by assessing the maximal oxygen consumption (VO2 max) during exercise, reflecting the body’s capacity to transport and utilize oxygen. Following a stroke, individuals often experience a decline in cardiorespiratory fitness due to factors such as reduced physical activity, muscle atrophy, weak and impaired motor function, cardiovascular deconditioning, impaired neural control, respiratory dysfunction, inflammation and oxidative stress, and metabolic changes [3,6,7,8]. After a stroke, cardiorespiratory fitness (CRF) is compromised with VO2peak values falling within the range of 8–22 mL/kg/min. This corresponds to 26% to 87%, respectively, of the CRF observed in healthy individuals matched for age and sex [9]. The assessment of cardiorespiratory fitness in stroke survivors is crucial for understanding their functional capacity and designing effective rehabilitation interventions [3,5].

Engaging in exercise and physical activity after a stroke plays a crucial role in diminishing the likelihood of subsequent cardiovascular events and recurrent strokes. Tailored exercise programs, including aerobic and strength training, have been demonstrated to enhance motor function, mobility, and cardiorespiratory fitness (CRF) in stroke survivors [10,11]. In addition, physical exercise also enhances the neuroplasticity process and it appears to be more robust during moderate to high-intensity exercise programs [12]. The intensity level has been identified as a crucial element in training adaptations, playing a role in sustaining and enhancing peripheral muscular oxidative capacity [13] as well as aerobic performance [14]. Previous literature reviews and meta-analyses already stated that aerobic training has benefits on post-stroke survivors’ CRF [15,16]. Furthermore, a recent review showed the benefits of high-intensity exercise on functional recovery, cardiovascular health, and neuroplasticity after stroke [17], and another meta-analysis by Luo et al. suggested that high-intensity exercise training could be specifically beneficial for cardiorespiratory fitness in stroke survivors [18].

A high-intensity exercise program includes both high-intensity training (HIT) and high-intensity interval training (HIIT). HIT features prolonged periods of vigorous exercise, while HIIT is characterized by brief episodes of maximum effort followed by intervals of lower-intensity exercise or rest. Additionally, other modalities such as high-intensity interval resistance training (HIIRT), high-intensity circuit training (HICT), and high-intensity functional training (HIFT) can be considered subgroups of these broader categories, incorporating elements of strength, endurance, and functional movements within the high-intensity framework [19]. Table 1 illustrates the classification of exercise intensities, based on guidelines from the American College of Sports Medicine. 

The designated intensity for high-intensity exercise should exceed 60% of the heart rate reserve (HRR) or VO2peak, surpass 70% of maximal heart rate, or a Borg Ratings of Perceived Exertion (RPE) scale score of 14 [20].

Heart Rate Reserve (HRR) is defined as the gap between a person’s maximum heart rate and their resting heart rate, and it is used to determine appropriate exercise intensity [20]. Similarly, VO2 Reserve (VO2R) quantifies the disparity between maximum oxygen consumption (VO2max) and resting oxygen consumption (VO2rest), providing a tailored approach to improve cardiovascular fitness [21]. VO2max is the maximum rate of oxygen consumption measured during incremental exercise, indicating the highest capacity of an individual’s aerobic energy system and considered the gold standard for assessing cardiovascular fitness. In contrast, VO2peak represents the highest rate of oxygen consumption measured during an exercise test, regardless of whether a plateau in oxygen uptake is achieved, reflecting an individual’s aerobic capacity under specific test conditions and not reflecting the subject’s absolute maximum capacity [20,22]. VO2max is often used in research and clinical settings wherein precise measurement of aerobic capacity is required. VO2peak, instead, is used in situations wherein it may not be feasible to push subjects to their absolute maximum (e.g., with certain populations like the elderly or those with health conditions). Additionally, the Rating of Perceived Exertion (RPE) scale is a subjective measure that assesses exercise intensity based on how hard an individual feels they are working, with a typical range from 6 to 20, wherein higher numbers indicate higher perceived exertion [23].

In detail, the study by Luo et al. indicated that high-intensity exercise (70–85% HRR/VO2peak, 3 to 5 times per week, for 30 to 40 min per session over 8 to 12 weeks) can enhance peak oxygen consumption (VO2peak) and 6 min walk distance in stroke survivors [18]. However, a recent study found that, while early initiation to home-based high-intensity interval training (HIIT) increased time spent in vigorous-intensity activities post-stroke, it did not lead to sustained improvements in long-term cardiorespiratory fitness, with activity levels declining to baseline at 12 months follow-up, highlighting the challenge of maintaining physical activity post-stroke and the need for strategies to support long-term adherence [24]. Moreover, the timing and intensity of exercise interventions in stroke rehabilitation vary significantly across studies, with some evidence suggesting that more intense and earlier interventions can improve motor outcomes, though challenges remain in the consistent implementation of high-intensity therapies [25].

Balance, another of the most impaired functions after a stroke, is a multifaceted neuromotor ability that revolves around the interplay of external forces exerted on our body, particularly on the spine, and the subsequent muscular reactions of the core. To attain and uphold balance, a range of sensorimotor adaptations are necessary to uphold a steady upright stance, whether in static or dynamic postures [26,27]. Individuals who have suffered a stroke frequently encounter sensory, cognitive, and motor aftereffects, which may result in challenges maintaining balance while standing or engaging in voluntary tasks, and responding effectively to prevent falls triggered by sudden postural disturbances [28]. Moreover, merely 30–50% of individuals affected by a stroke achieve community ambulation, a crucial metric within the activities and participation domains outlined in the International Classification of Functioning, Disability, and Health (ICF) [29,30]. Autonomous walking serves as a significant marker of overall independence and quality of life, constituting one of the primary objectives in stroke rehabilitation efforts [31,32].

To date, many different therapeutic approaches have been investigated in improving both balance and walking in post-stroke patients. In particular, the literature supports physical therapy, including a combination of conventional rehabilitation exercises and gym-based interventions such as aerobic exercises training [33], exercises like the sit-to-stand combined with real-time visual feedback [34], treadmill training and treadmill with functional electrical stimulation [35], but also vibration therapy, rhythmic auditory stimulation training, boxing therapy [36], dual-task training [37], non-invasive brain stimulation/spinal cord stimulation [38], mirror therapy with afferent electrical stimulation [39], and the use of ankle–foot orthosis (AFO) [40]. Virtual reality treatment is still debated [41,42,43]. To the best of our knowledge, just a few studies have investigated so far the impact of high-intensity exercise training on gait and balance in post-stroke survivors, with controversial results [19,20,21]. While high-intensity interval training (HIIT) has shown greater improvements in cardiorespiratory fitness compared to moderate-intensity continuous training (MICT), another recent study found no significant differences between HIIT and MICT in terms of balance or walking distance [22].

Finally, an interesting recent study found that individuals recovering from a stroke perceived higher-intensity training as a factor that promotes, rather than hinders, their involvement in exercise rehabilitation. These results have the potential to question preconceived notions regarding the impact of exercise intensity on participant engagement [23].

Overall, to date, there is limited evidence and no consensus among specialists about the best rehabilitation protocol to improve CRF, walking, and balance in stroke survivors and, particularly, about the efficacy of high-intensity exercise training on these outcomes. Thus, we aimed to investigate specific and different high-intensity exercise training protocols on cardiorespiratory fitness together with its impact on gait ability and balance in stroke survivors. These aspects hold paramount importance in this population, given their frequent impairment, which profoundly impacts the daily lives of the affected individuals.

## 2. Materials and Methods

### 2.1. Registration

This systematic review and meta-analysis were performed ethically in accordance with the Preferred Reporting Items for Systematic Review and Meta-Analyses (PRISMA) statement. The protocol was registered in PROSPERO (registration number CRD42023456773).

### 2.2. Outcomes Measures

Cardiorespiratory fitness (CRF) can be assessed through a variety of direct and indirect methods. Direct methods, such as measuring peak oxygen uptake (VO2max) and lactacidemia, are considered the gold standard for accurately evaluating an individual’s CRF. Indirect methods, including the 6 min walk test (6 MWT), the 10 m walk test (10 MWT), and maximal aerobic speed tests, are also commonly used. It is important to note that certain tests, such as the 6 MWT, are specifically tailored for particular groups, such as individuals with limited mobility or those recovering from stroke, offering a practical alternative in clinical settings [44].

The main outcome measures considered in the studies included in this review were the VO2 peak, which is utilized in scenarios wherein achieving maximum exertion is not feasible, and the 6 MWT, as clinical measures of the CRF. As secondary outcomes, we also included studies evaluating the 10 m walking test (10 MWT), the Berg Balance Scale (BBS), and the Timed Up and Go (TUG) test to obtain information on walking and balance as well.

The 6-Minute Walk Test (6 MWT) evaluates aerobic capacity and endurance by measuring the distance a person can walk on a flat, hard surface within six minutes. The 10-Meter Walk Test evaluates walking speed over a short distance, typically used to assess gait and mobility. The Berg Balance Scale is a 14-item assessment measuring balance through tasks such as standing, turning, and reaching, helping to predict fall risk. The Timed Up and Go (TUG) test involves timing an individual as they stand up from a chair, walk three meters, turn around, walk back, and sit down, providing insight into mobility, balance, and risk of falling [45,46,47,48].

### 2.3. Search Strategy

Two investigators independently examined and systematically searched Pubmed/Medline, Cochrane, EBSCO, EMBASe, and Scopus for RCTs published up to 31 May 2023. Each source was searched on the same date, with the last search performed at the end of May 2023. A third author solved the disagreements between the investigators. A comprehensive search strategy was employed across multiple databases to identify relevant studies on the effects of high-intensity exercise in post-stroke survivors. The search included a combination of key terms related to the population (e.g., “stroke”, “cerebrovascular accident”, “ischemic stroke”), the intervention (e.g., “high-intensity exercise”, “aerobic interval training”), the comparator (e.g., “moderate exercise”, “continuous moderate physical activity”), and outcomes (e.g., “cardiorespiratory fitness”, “balance”). Boolean operators (AND, OR) were used to combine these terms, and the search was conducted in PubMed, Cochrane, EBSCO, EMBASE, and Scopus. The complete search strategies tailored to each database are available as a Supplementary File in Supplementary Figures S1 and S2.

### 2.4. Selection Strategy

In order to provide a comprehensive overview of the topic, our inclusion criteria intentionally avoided excessive specificity. We aimed to encompass a broad range of relevant research involving high-intensity exercise in post-stroke survivors, recognizing the inherent diversity in the available studies. Following the PICO model [49], we considered eligible RCTs satisfying the following criteria:

(P) Participants: first stroke-survivors, age ± 18 years.

(I) Intervention: high-intensity physical exercise rehabilitation program, in accordance with the definition of high-intensity exercise provided by the American College of Sports Medicine classification of exercise relative intensity mentioned above. More in detail, for each treatment should be clearly described the type of exercise, the frequency, the duration of a single session, the duration of the whole treatment, and the intensity.

(C) Comparator: low-to-mild physical exercise rehabilitation program or no exercise.

(O) Outcome: the primary outcomes were changes in the CRF of the patients assessed through the Six-Minutes Walking Test (6 MWT) and the maximal oxygen uptake (VO2peak). The secondary outcomes instead were the 10-Meter Walk Test (10 MWT), the Berg Balance Scale (BBS), and the Timed Up and Go test (TUG).

We included RCTs that were peer-reviewed, published in English language, and appeared in journals that met the criteria for international reach, including a global scope of authorship, wide readership, rigorous peer-review, indexing in major international databases (e.g., PubMed or Scopus), and an internationally diverse editorial board.

The exclusion criteria were as follows: (i) studies involving animals; (ii) language other than English; (iii) conference abstracts and master or doctorate thesis; (iv) participants with pregnancy, clinical instability, severe orthostatic hypotension, or other clinical conditions preventing them from performing physical activity; (v) patients affected by moderate-to-severe cognitive impairment (MMSE ≤ 24); (vi) patients unable to walk or requiring assistive device to walk; (vii) patients with a body mass index ≥ 31; (viii) participants already enrolled in other studies; (ix) patients who had already undergone a specific rehabilitation treatment after stroke.

After eliminating duplicates, two investigators (M.B.B. and M.B.) separately examined the titles and abstracts of the retrieved articles to pinpoint relevant ones. In cases of discrepancies, they resolved them through collaborative discussion. If an agreement could not be reached, a third investigator (A.B.) was consulted. Furthermore, reference lists from the included studies were examined to identify additional relevant records. Finally, two investigators (M.B.B. and M.B.) assessed the full text of the relevant records, and any disagreements were resolved by involving a third reviewer (A.B.).

### 2.5. Data Extraction and Synthesis

Each full-text document underwent a rigorous evaluation for eligibility, conducted by two investigators working independently from each other (M.B.B. and M.B.). Relevant data were extracted using Excel, and any disparities in their assessments were resolved through discussion between the two reviewers or by seeking input from a third investigator (A.B.). This process was entirely manual, without the use of automated tools.

The following information were extracted: (1) author details; (2) journal information; (3) publication year; (4) nationality; (5) age of study participants; (6) intervention characteristics; (7) comparison group characteristics; (8) mean time from stroke; (9) type of stroke; (10) localization of stroke; (11) outcome measures; (12) main findings.

### 2.6. Quality Assessment and Risk of Bias

Two independent reviewers (S.S. and F.S.) assessed the risk of bias using Version 2 of the Cochrane risk-of-bias tool for randomized trials (RoB 2) [50]. When discrepancies arose, they were resolved through discussion or by consulting a third reviewer (S.L.). Each domain of RoB 2 was evaluated, including random sequence generation, allocation concealment, blinding of participants and personnel, blinding of outcome assessment, incomplete outcome data, selective outcome reporting, and other sources of bias. Each domain was rated as low, high, or unclear risk, as shown in Supplementary Table S1.

### 2.7. Statistical Analysis

The DerSimonian and Laird method was used to calculate the random-effects meta-analytic estimate of the difference between means (pMD) for pre–post intervention variations in Berg Balance Scale (BBS) and tests including the Timed Up and Go (TUG), 6 min walking test (6 MWT), 10 m walking test (10 MWT), and VO2 values between the experimental and control groups. The Hartung–Knapp–Sidik–Jonkman method was used to calculate the 95% confidence interval (95%CI) given the unequal sample size of the included studies.

For this purpose, the differences between the means of the measurements calculated at the end of the follow-up (post) and at the beginning of the study (pre) were computed separately for the intervention and control groups in each study.

Additionally, standard deviations of post–pre variations were calculated, taking into account the within-patient correlation as follows:

(1) $$\sqrt{{var}_{pre}+{var}_{post}-2*\rho *{SD}_{pre}*{SD}_{post}}$$

where *var_pre_* and *var_post_* are the variances of the means of parameters measured at the beginning of the study and at the end of the follow-up, *SD_pre_* and *SD_pos_* are the corresponding standard deviations, and *ρ* represents the correlation coefficient between pre and post measurements. The value of *ρ* was set at 0.6. Once the pre–post differences were calculated separately for the two arms, the difference between the means of pre–post in the two groups and the corresponding standard error were computed.

The presence of heterogeneity among studies was tested using Cochran’s Q test and quantified using the I^2^ index. I^2^ values > 50% indicate the presence of heterogeneity. When multiple intervention arms were present in the same trial, the less effective one was considered in the main analysis, and subsequently, a sensitivity analysis was performed considering the most effective intervention arm. Additionally, meta-analytic estimates were calculated considering the variation between baseline and the end of follow-up.

In addition to the main analysis, a stratified analysis was conducted for the treatment duration. Analyses were performed when at least one of the strata had at least three estimates available.

The presence of publication bias was assessed using a forest plot and Egger’s test.

## 3. Results

### 3.1. Search Results

In total, 1220 records were initially sourced from the five databases examined. After removing duplicates from the initial pool of records, 1162 studies were subjected to eligibility assessment and reviewed based on their titles and abstracts. Consequently, 1126 records were eliminated, and 36 studies underwent a detailed full-text screening. Ultimately, 11 articles were excluded due to ineligibility (nine were not RCTs, two reported insufficient data); meanwhile, 25 randomized controlled trials (RCTs) met the criteria for inclusion in this systematic review.

**Figure 1:**
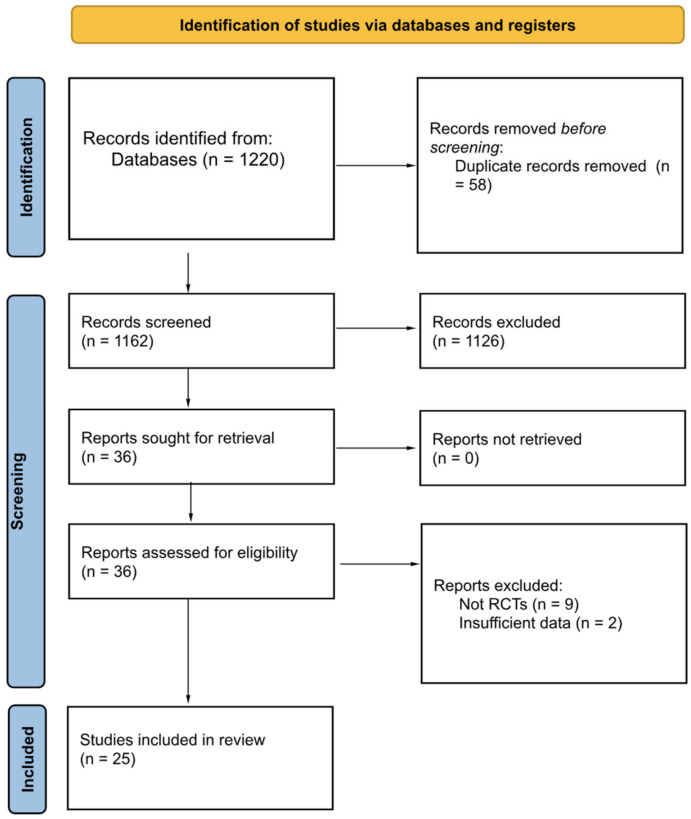
PRISMA 2020 flow diagram for new systematic reviews.

The search process is outlined in detail in the PRISMA flow diagram (see Figure 1). Therefore, the present systematic review includes the 25 following RCTs: Ahmed [51], Boyne [52,53]; Gjellesvik [54,55]; Globas [56]; Holleran [57]; Hornby [58,59]; Ivey [60,61,62]; Jin [63]; Lamberti [64]; Lapointe [65]; Leddy [66]; Lee [67]; Linder [68]; Macko [69]; Munari [70]; Pang [71]; Reynolds [72]; Severinsen [73]; Tang [74,75].

### 3.2. Characteristics of Included RCTs and Participants

The RCTs included in the present review were published between 2005 [69,71] and 2023 [53,65] and were conducted in the United States of America [*n* = 12 [52,53,57,58,59,60,61,62,66,67,68,69]], Germany [*n* = 1 [56]], Italy [*n* = 2 [64,70]], China [*n* = 1 [63]], Canada [*n* = 4 [65,71,74,75]], Denmark [*n* = 1 [73]], Norway [*n* = 2 [54,55]], Australia [*n* = 1 [72]], and Pakistan [*n* = 1 [51]].

The sample size of the RCTs included ranged from 12 [57] to 133 [63], the 25 RCTs described results for a total of 1229 participants [758 (61.7%) males]. The mean age ranged from 55 [57] to 69 [56,65], with a mean age of 60 years. The mean time since stroke at recruitment ranged from two weeks [75] to 5.8 years [70].

The sample characterization of each study included in the present review is summarized in detail in Table 2.

### 3.3. Quality Assessment and Risk of Bias

**Figure 2:**
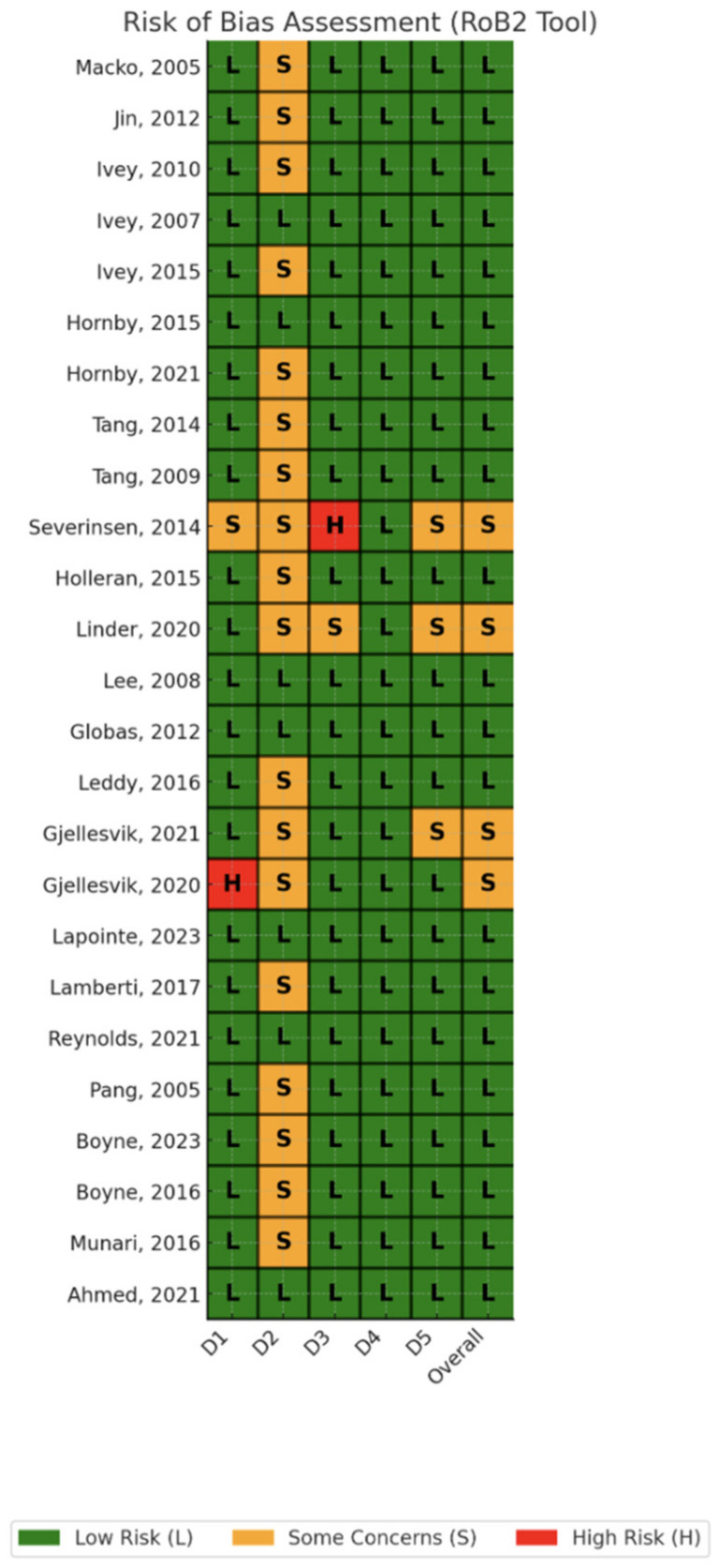
Risk of bias assessment [51,52,53,54,55,56,57,58,59,60,61,62,63,64,65,66,67,68,69,70,71,72,73,74,75].

The quality of the included studies was assessed using the Cochrane Risk of Bias 2 (RoB2) tool for randomized trials. Among the randomized trials, the studies exhibited varying levels of bias across different domains. While some studies demonstrated a low risk of bias in certain areas, others showed higher risks, particularly in the domain concerning deviations from intended interventions. Consequently, these studies were judged to have some concerns regarding bias. Although the evidence was considered adequate to support preliminary conclusions, it should be interpreted with caution due to the identified risks, as detailed in Figure 2.

### 3.4. Interventions

The RCTs included in the present systematic reviews reported high heterogeneity in the type of intervention treatments performed. The most frequently studied high-intensity exercise modalities were treadmill, which was figured in nine studies [52,54,55,56,60,61,62,66,69], and overground walking plus treadmill which was present in three studies [53,57,58]. All the other RCTs provided different types of intervention treatments (each of them is detailed in Table 3).

Analyzing the single-session duration, seven studies showed a session duration of less than 40 min [52,54,62,65,67,72,74], eight studies between 40 and 59 min [51,53,57,60,61,63,69,70], and eight studies had a session duration of more than 60 min [58,59,64,66,68,71,73,75]. One study had a session duration between 30 and 50 min [47] and in one this parameter was not specified [55]. The mean single-session duration of all the included 25 RCTs was 48 min.

Moreover, one RCT showed a weekly frequency of one or two sessions per week [72], nineteen studies a frequency of three or four sessions per week [52,53,54,55,56,57,60,61,64,65,66,67,68,69,70,71,73,74,75], and two studies five sessions or more per week [51,63]. The mean weekly frequency among all studies was 3.4.

The total program length was 3 months or less in seventeen studies [51,52,53,54,55,57,58,59,63,64,66,67,68,70,72,73,74], between 3 and 6 months in three studies [56,69,71], and 6 months or more in five studies [60,61,62,65,75]. The mean program length was 13.4 weeks. Finally, the setting was, for all the studies, an outpatient one, except for one study [74], for which it was an inpatient setting.

Information about the intervention is fully reported in Table 3.

### 3.5. Main Findings

All the results concerning outcome measures are comprehensively detailed in Table 4.

#### 3.5.1. Primary Outcomes

The primary outcome assessed in this review was the cardiorespiratory fitness (CRF), measured through the 6 MWT and the VO2peak.

The meta-analysis revealed a significant improvement in the 6 MWT performance in the high-intensity exercise group compared to the control group, with a mean difference of 88.87 m (95% CI 28.08; 148.67), though high heterogeneity (I^2^ = 96%) suggests variability in the results across studies.

The 6 MWT was conducted in nineteen of the twenty-five included studies [52,53,62,63,64,68,69,70,71,72,73,74,75], as fully reported in Table 4.

**Figure 3:**
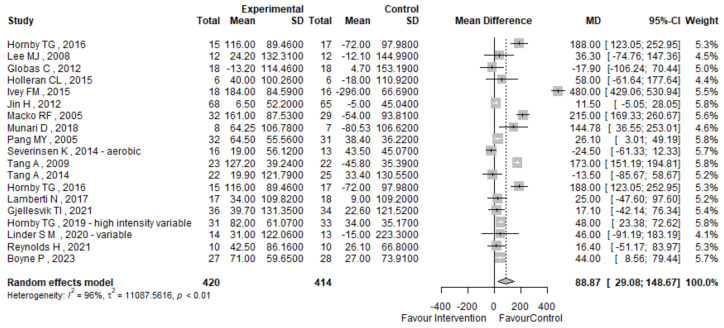
The 6 MWT forest plot [53,55,56,57,58,59,62,63,64,67,68,69,70,71,72,73,74,75].

Figure 3 shows the 6 MWT forest plot, which details the studies’ specific mean difference (MD), the corresponding 95% confidence interval (CI), and heterogeneity evaluation. The reported results show that pre–post intervention variations in the 6 MWT, calculated by the random-effects meta-analytic estimate of the difference between means, are higher in patients treated in the experimental group (high-intensity exercise training) rather than in the control group (low-to-mild exercise or no exercise) with a mean difference of 88.87 m (95% CI 28.08; 148.67), suggesting a higher improvement in post-treatment 6 MWT results in the interventional group compared to the control one.

However, our results also highlight an I^2^ heterogeneity index of 96%, with a *p*-value < 0.01, thus deserving a more detailed investigation. Given that, in some studies [32,53,59,68,73], multiple treatment arms and outcome assessment strata were defined, a sensitivity analysis of the intervention arm with greater efficacy was conducted to understand its burden in the study and also an analysis stratified by treatment duration, as shown in Supplementary Table S2.

**Figure 4:**
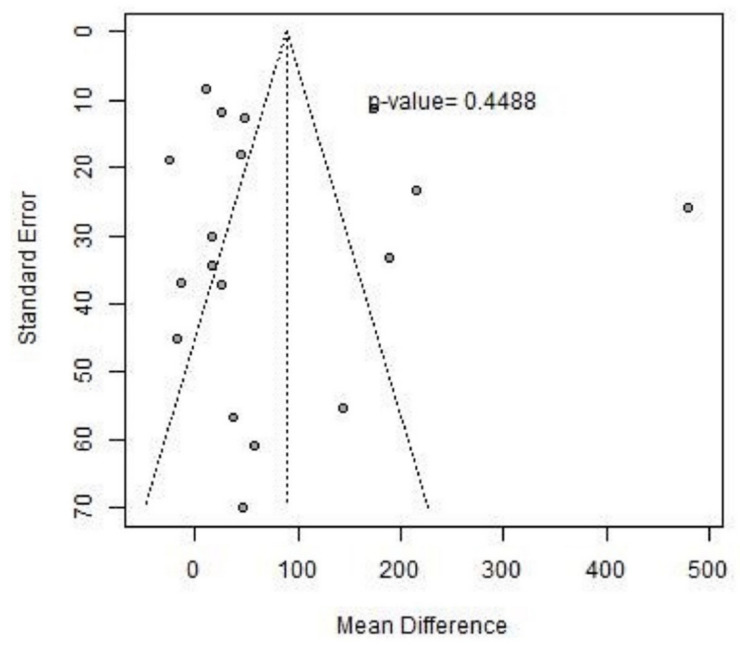
The 6 MWT funnel plot.

The presence of publication bias was verified by a funnel plot and by calculating the *p*-value using Egger’s test, which turned out to be >0.10, the reference value below which the *p*-value of Egger’s test considers significant the asymmetry of the distribution in the funnel plot. Figure 4 shows the 6 MWT funnel plot.

The analysis also demonstrated a notable increase in VO2peak for the high-intensity exercise group, with a mean difference of 4.13 mL/kg/min (95% CI 2.44; 5.82), indicating enhanced cardiorespiratory fitness, although substantial heterogeneity (I^2^ = 97%) and variability in study designs warrant further scrutiny.

**Figure 5:**
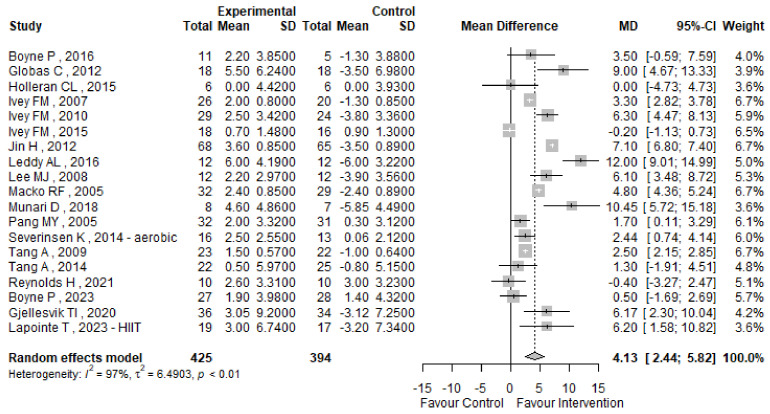
VO2peak forest plot [52,53,54,56,57,60,61,62,63,65,66,67,69,70,71,72,73,74,75].

**Figure 6:**
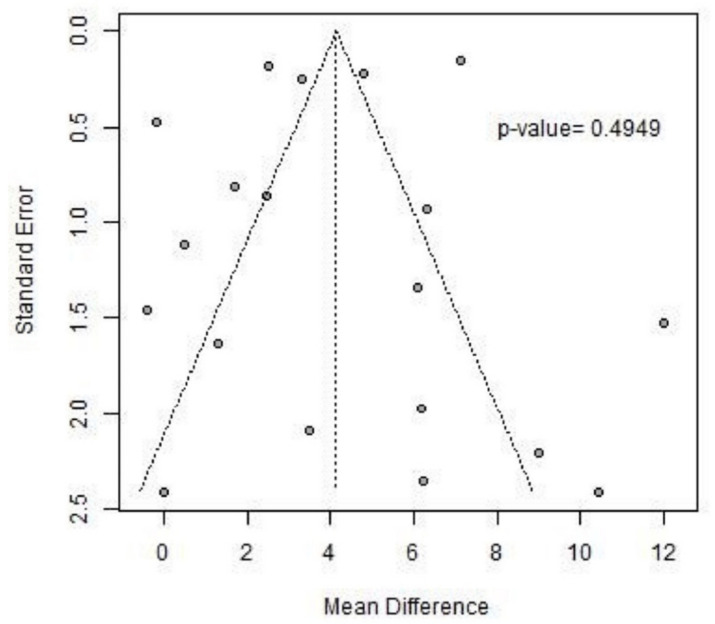
VO2peak funnel plot.

VO2peak was evaluated across nineteen studies included in our analysis [52,53,54,56,57,60,61,62,63,65,66,67,69,70,71,72,73,74,75], as detailed in Table 4. Figure 5 depicts in detail the forest plot for the VO2peak, delineating the specific mean difference of each study, along with the corresponding 95% confidence intervals and heterogeneity assessment. Our findings reveal a significant mean difference (MD) of 4.13 mL/kg/min (95% CI 2.44; 5.82) in VO2peak between pre and post-intervention measurements, favoring individuals in the experimental group over the control group. This suggests an improvement in VO2peak following treatment within the experimental group. Nevertheless, we observed considerable heterogeneity, with an I^2^ index of 97% and a *p*-value < 0.01, warranting further investigation. In light of potential confounders such as multiple treatment arms and varying outcome assessment strata in some studies [32,65,73], we conducted sensitivity analyses focusing on the intervention arm with superior efficacy and stratified by treatment duration, as detailed in Supplementary Table S2. Assessment for publication bias utilizing Egger’s test yielded a non-significant *p*-value of 0.4949, indicating no evidence of asymmetry in the funnel plot, as illustrated in Figure 6.

#### 3.5.2. Secondary Outcomes

Secondary outcomes were evaluated to provide insights into the effects of interventions on functional performance and balance. The findings from the 10-Meter Walk Test (10 MWT), Berg Balance Scale (BBS), and Timed Up and Go (TUG) test, which are summarized in Table 4, reveal varying impacts and highlight the need for further investigation due to observed heterogeneity and variability.

**Figure 7:**
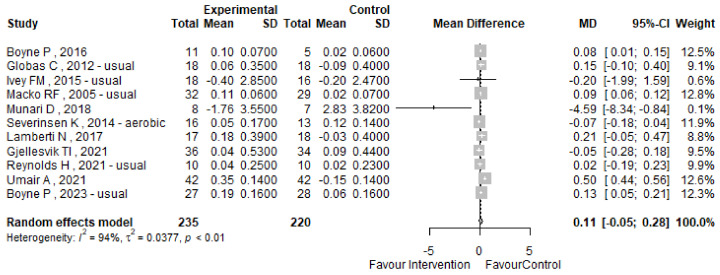
The 10 MWT forest plot [51,52,53,55,56,62,64,69,70,72,73].

**Figure 8:**
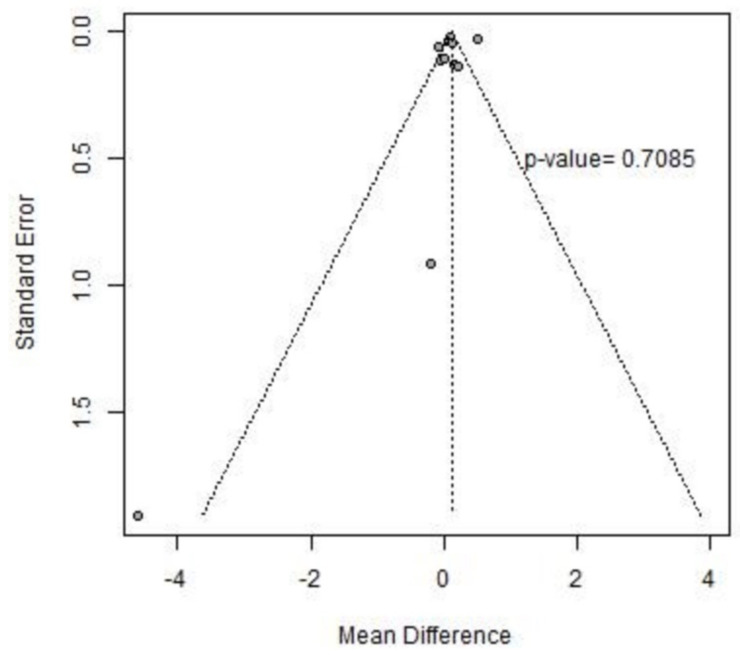
The 10 MWT funnel plot.

The 10 MWT (10-Meter Walk Test) was evaluated across eleven studies included in our analysis, as detailed in Table 4. Figure 7 presents the forest plot illustrating the mean difference (MD) in the 10 MWT, along with the corresponding 95% confidence intervals and heterogeneity assessment. Our analysis indicates a modest mean difference (MD) of 0.11 s (95% CI −0.05; 0.28) in the 10 MWT between pre and post-intervention phases. However, significant heterogeneity was observed, with an I^2^ index of 94% and a *p*-value of 0.01, suggesting the need for further investigation. Notably, studies with multiple treatment arms and layers require sensitivity analysis to delineate potential sources of variability, as detailed in Supplementary Table S2. Evaluation for publication bias via Egger’s test yielded a non-significant *p*-value of 0.7085, indicating no apparent asymmetry in the funnel plot, as shown in Figure 8.

**Figure 9:**
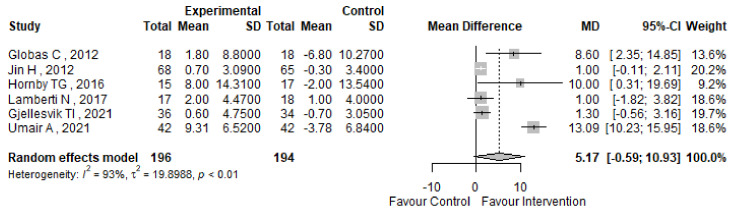
BBS forest plot [51,55,56,58,63,64].

**Figure 10:**
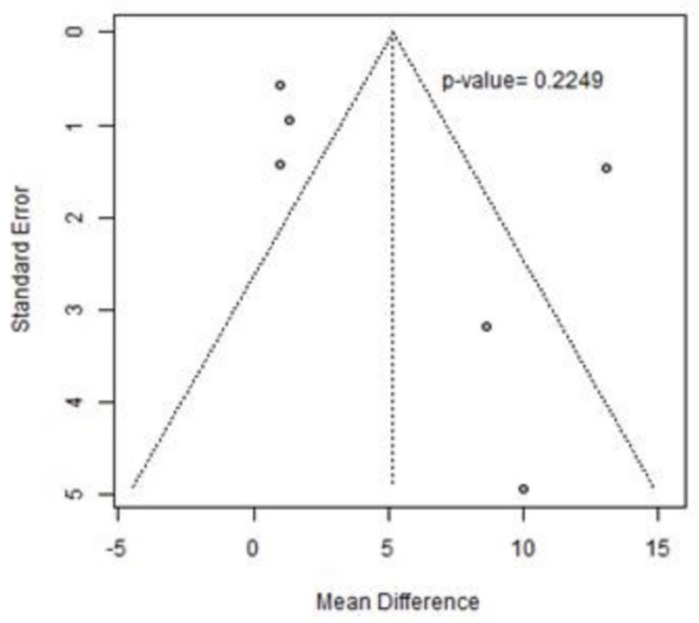
BBS funnel plot.

The Berg Balance Scale (BBS) was assessed across six studies included in our analysis [51,55,56,58,63,64], as reported in Table 4. Figure 9 illustrates the forest plot representing the mean difference (MD) in BBS scores, along with the corresponding 95% confidence intervals and heterogeneity assessment. Our analysis reveals an MD of 5.17 points (95% CI 0.59; 10.93) in BBS scores between pre and post-intervention phases; however, the improvement following treatment is not statistically significant. In addition, substantial heterogeneity was observed, with an I^2^ index of 93% and a *p*-value of less than 0.01, necessitating further scrutiny. Remarkably, none of the studies in the forest plot employed multiple treatment layers in terms of exercise modality or duration, obviating the need for sensitivity analysis. The funnel plot interpretation suggests no evidence of publication bias, corroborated by Egger’s test with a non-significant *p*-value of 0.2249 (Figure 10).

**Figure 11:**
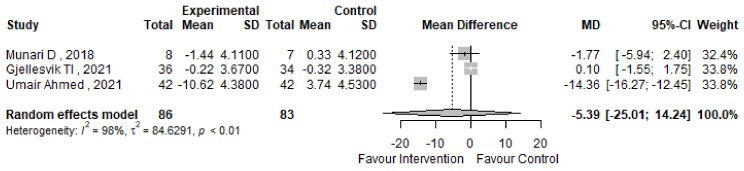
TUG forest plot [51,55,70].

Finally, the Timed Up and Go (TUG) test was unfortunately assessed in only three studies included in our meta-analysis [51,55,70], as reported in Table 4. Regrettably, the TUG test did not yield statistically significant results regarding the most effective training regimen. Despite this, the mean difference in TUG scores between pre and post-intervention phases was −5.39 s (95% CI −25.01; 14.24), suggesting some variability in outcomes, as shown in Figure 11. Given the limited number of studies, further analysis and drawing definitive conclusions may be challenging.

## 4. Discussion

High-intensity exercise drives several key physiological adaptations that significantly impact recovery in stroke rehabilitation. Firstly, it enhances muscle oxidative capacity, which allows for better energy production and endurance. This is achieved through an increase in mitochondrial density and efficiency, enabling muscles to utilize oxygen more effectively. Secondly, high-intensity training boosts capillary density, improving blood flow and nutrient delivery to muscle tissues. Additionally, it enhances neuromuscular efficiency by promoting neural plasticity, which strengthens the connection between the brain and muscles, improving coordination and motor control. These adaptations collectively lead to improved VO2peak, better cardiovascular health, and enhanced gait and balance abilities. Understanding these mechanisms provides a foundation for designing targeted rehabilitation programs that maximize functional recovery post-stroke [3,6,76].

This systematic review aimed to evaluate the impact of high-intensity training on cardiorespiratory fitness, walking, and balance among stroke survivors, filling a gap in the current literature where consensus on optimal rehabilitation protocols remains elusive. Twenty-five randomized controlled trials (RCTs) met our inclusion criteria, conducted primarily in outpatient settings across various countries. These studies, spanning from 2005 to 2023, enrolled a total of 1229 participants with an average age of 60 years and a mean time since stroke ranging from two weeks to 5.8 years. Notably, the interventions exhibited high heterogeneity, with the most common modalities being treadmill-based exercises. While the primary outcomes, including the Six-Minute Walk Test (6 MWT) and peak oxygen uptake (VO2peak), demonstrated significant improvements post-intervention in the high-intensity training groups, substantial heterogeneity was observed, warranting further investigation. Secondary outcomes, such as the 10-Meter Walk Test (10 MWT), Berg Balance Scale (BBS), and Timed Up and Go (TUG) test, also exhibited varying degrees of improvement, though with notable heterogeneity across studies. Despite limitations, this review underscores the potential efficacy of high-intensity exercise training in enhancing CRF, gait ability, and balance in stroke survivors, offering insights for future research and clinical practice.

### 4.1. Effects on CRF

Our review offered additional validation of the beneficial impact of high-intensity training exercises on enhancing cardiorespiratory fitness among individuals recovering from stroke.

Firstly, our analysis underscored the markedly low baseline levels of CRF observed in stroke survivors, in accordance with previous studies [9,18].

Specifically, the baseline levels of peak oxygen uptake (VO2peak) were below 18 mL/kg/min in 13 out of 19 studies included in our review. Such diminished CRF levels are indicative of compromised functional capacity, with VO2peak values falling below the normal range for older individuals. Moreover, the baseline levels of the Six-Minute Walk Test (6 MWT) were consistently below 300 m in 10 out of the 19 studies examined. These findings emphasize the substantial impact of stroke on the physical health and functional abilities of the affected individuals, highlighting the urgent need for targeted rehabilitation interventions to address these deficits and enhance overall well-being.

Considering the intervention efficacy, we found that post-treatment 6 MWT and VO2peak results showed higher improvement in the experimental group with a mean difference of 88.87 m (95% CI 28.08; 1480.67) and of 4.13 mL/kg/min (95% CI 2.24; 5.48), respectively, in accordance to the results published by Luo et al. in 2020 [15]. The 6 min walk test (6 MWT), assessing both aerobic capacity and gait ability, emerges as a crucial predictor of family and community walking engagement, surpassing gait speed in its predictive power. Moreover, it exhibits a significant correlation with the Stroke Impact Scale score, suggesting that enhancements in the 6 MWT may lead to heightened participation and improved activities of daily living among stroke survivors [77,78].

Expanding upon Luo et al.’s research, which primarily focused on cardiorespiratory fitness (CRF) outcomes such as peak oxygen consumption (VO2peak) and the 6-Minute Walk Test (6 MWT) across 17 randomized controlled trials (RCTs) involving 707 patients, our review serves two main purposes. Firstly, we aim to scrutinize recent findings post-2020 and extend the investigation beyond CRF, encompassing additional critical aspects such as walking ability and balance. This decision stems from the positive outcomes of high-intensity interval training (HIT) reported by Luo et al. [18], prompting our study to involve nearly double the number of participants (1229 vs. 707) to bolster the evidence of HIT’s efficacy on CRF. Furthermore, our exploration seeks to provide fresh insights into the interplay between CRF, walking, and balance, thus broadening our understanding of the multifaceted effects of HIT.

### 4.2. Effects on Gait Ability and Balance

Our findings concerning the 6-Minute Walk Test (6 MWT) are in concordance with a recent systematic review conducted by Mah et al. in 2022, which underscores the efficacy of high-intensity training in enhancing performance in lower-limb functional tasks, particularly evident in the 6 MWT and gait speed [37]. However, divergent outcomes surfaced concerning the 10 MWT, where we noted no significant effects of high-intensity training, potentially attributed to the limited number of studies available, comprising only three. Mah’s systematic review primarily focused on acute and sub-acute post-stroke patients, delineating lower-limb function as a representation of post-stroke impairments, with outcome measures including the 6 MWT, gait speed, steps per day, Berg Balance Scale (BBS), and Barthel index (BI). Discrepancies observed between our findings and Mah’s review could be attributed to variances in the studied populations (our study predominantly involved chronic post-stroke patients), alongside high heterogeneity and limited study numbers.

Interestingly, our study also investigated the potential effectiveness of HIT in enhancing balance, as measured by the BBS. Our results do not reveal a statistically significant association between HIT and improvements in BBS scores, which is in line with the findings reported by the systematic review of van Duijnhoven et al. This review included 28 studies (985 participants) and evaluated the BBS as an outcome. Their subgroup analyses conducted on studies with BBS outcomes revealed a notable enhancement following balance and/or weight-shifting training, with an increase of 3.75 points (+6.7%; 95% CI, 1.71–5.78; *p* < 0.01; I^2^ = 52%). Similarly, gait training resulted in a significant improvement of 2.26 points (+4.0%; 95% CI, 0.94–3.58; *p* < 0.01; I^2^ = 21%). Conversely, no significant effects were observed for alternative training protocols, including high-intensity aerobic exercise training.

In contrast, the study by Moore et al. compared the impact of high-intensity stepping intervention with usual care on stepping activity, walking, and balance outcomes among an inpatient stroke population. The high-intensity stepping intervention group exhibited a statistically significant and clinically meaningful enhancement in self-selected and fastest gait speeds compared to usual care. Moreover, changes in BBS and 6 MWT scores were also found to be significantly and clinically different between the two groups. These findings suggest that implementing high-intensity stepping training during inpatient rehabilitation could lead to superior walking and balance outcomes among stroke patients [79].

### 4.3. Practical Recommendations

Finally, it is crucial to keep in mind that physical impairments resulting from stroke can impede stroke survivors from achieving and maintaining high-intensity exercise regimens [80,81], underscoring the significance of tailored high-intensity training interventions for this population. The recent literature suggests that modalities like high-intensity interval training (HIIT) may be more feasible for stroke survivors [82]. Such interventions have the potential to address barriers faced by stroke survivors, including central and local fatigue, the mechanisms of which remain incompletely understood [83], facilitating the attainment of the necessary exercise intensity for maximizing functional improvements [37].

Based on the evidence from the included RCTs and the findings of our systematic review, we present preliminary suggestions for high-intensity exercise training in stroke rehabilitation. These suggestions reflect the diverse interventions studied and are intended to offer a general framework. However, due to variability in patient characteristics such as age, gender, stroke severity, and affected body parts, it is essential that each training program be carefully tailored to the individual needs of the patients. Training sessions should ideally last around 48 min, as this was the mean duration observed across the studies. Sessions should be conducted at least three times per week, with the mean frequency being 3.4 times per week, to ensure consistent improvements. Programs should generally span approximately 13 weeks to maximize benefits. Common and effective modalities include treadmill training and a combination of overground walking plus treadmill exercises, which can be adapted according to the disability and residual capabilities of patients. To maximize benefits, professionals should incorporate exercises tailored to the individual capabilities. Finally, considering the professionals involved, while kinesiologists are essential for general fitness and exercise prescription, the complex needs of stroke survivors may sometimes require the specialized clinical expertise of physiotherapists. Therefore, we opted not to focus on and identify a particular professional (kinesiologist or physiotherapist), as the appropriate choice can vary depending on the setting and context. Anyhow, it is imperative to conduct further meticulous investigation in this research domain to identify the most effective and safe high-intensity training modality for stroke survivors, aiming to enhance their fitness levels and motor abilities.

### 4.4. Limitations

The systematic review and meta-analysis presented here are subject to several limitations that warrant consideration. Firstly, the included studies exhibited significant heterogeneity in the type, frequency, duration, and intensity of the high-intensity exercise interventions, which may have contributed to variability in treatment effects across studies and limited our ability to determine optimal exercise protocols. Secondly, variations in outcome measures and assessment protocols among studies may have influenced the observed treatment effects, making it challenging to draw definitive conclusions. Additionally, the sample sizes within individual studies varied, and the majority were conducted in high-income countries, potentially limiting the generalizability of our findings to more diverse populations. Despite efforts to minimize bias, some degree of bias may still exist within individual studies, particularly related to blinding and selective outcome reporting. Furthermore, our analysis may be vulnerable to publication bias, since studies with positive outcomes are more likely to be published, which could potentially distort the overall results. Moreover, the lack of long-term follow-up assessments in many of the included studies limits our understanding of the sustainability of improvements in cardiorespiratory fitness, gait ability, and balance over time. Finally, there may be other unmeasured variables, such as concurrent rehabilitation therapies, lifestyles, or comorbidities, which could have influenced the observed treatment effects.

Acknowledging these limitations is crucial for interpreting the findings of our study and guiding future research efforts aimed at optimizing high-intensity training interventions for stroke survivors. Further well-designed RCTs with larger sample sizes, standardized protocols, longer follow-up durations, and diverse participant populations are needed to address these limitations and provide more robust evidence for clinical practice.

## 5. Conclusions

In conclusion, our systematic review highlights the promising impact of high-intensity exercise interventions on enhancing cardiorespiratory fitness (CRF) and balance among stroke survivors. Meta-analyses of twenty-five randomized controlled trials involving 1229 participants demonstrated significant improvements in CRF, as indicated by the Six-Minute Walking Test (6 MWT) and peak oxygen uptake (VO2peak), following high-intensity exercise interventions compared to control groups. However, no significant differences were observed in other measures of gait ability including 10 MWT and TUG and, similarly, no significant improvements were found in balance, as assessed by the BBS. These findings underscore the potential efficacy of high-intensity training in addressing CRF deficits in stroke survivors. Nevertheless, there remains a need for standardization of protocols and long-term follow-up studies to optimize rehabilitation outcomes in this population.

## Figures and Tables

**Table 1:** American College of Sports Medicine classification of exercise relative intensity.

Intensity	%HRR ^1^ or VO2R2	%peak VO2 ^2^	%max HR ^3^	RPE ^4^ Borg Scale
Very light	<20	<25	<35	10
Light	20–39	25–44	35–54	10–11
Moderate	40–59	45–59	55–69	12–13
Heavy	60–84	60–84	70–89	14–16
Very heavy	≥85	≥85	≥90	17–19
Maximal	100	100	100	20

^1^ HRR: heart rate reserve; ^2^ VO2R: VO2 reserve; ^3^ HR: heart rate; ^4^ RPE: rating of perceived exertion.

**Table 2:** Sample characteristics of the included studies.

Study	Country	N° Patients	N° Pts Intervention Group	N° Pts Control Group	Sex (Male)	Age (Years, Mean)	Time Since Stroke (Months, Mean)	Type of Stroke (Ischemic)	Lateralization of Stroke (Right)	BMI ^1^ (kg/m^2^, Mean)
Ahmed, 2021 [51]	PAK	84	42	42	37	62	7	51	48	26
Boyne, 2023 [53]	USA	55	27	28	36	62	30	24	28	28
Boyne, 2016 [52]	USA	16	11	5	9	58	50.4	11		27.8
Gjellesvik T, 2020 [54]	NOR	70	36	34	43	58	26	57	34	27
Gjellesvik, 2021 [55]	NOR	70	36	34	43	58	26	57	34	27
Globas, 2012 [56]	GER	36	18	18	29	69	64.8		13	26.4
Holleran, 2015 [57]	USA	12	6	6	7	55	34.8		4	
Hornby, 2016 [58]	USA	32	15	17	24	58	3.4	21	24	
Hornby, 2019 [59]	USA	97			58	58		66	50	
Ivey, 2007 [60]	USA	46	26	20		63				28
Ivey, 2010 [61]	USA	53	29	24	29	61				25
Ivey, 2015 [62]	USA	34	18	16	21	62				26
Jin, 2012 [63]	CHI	133	68	65	94	57	18	59	69	
Lamberti, 2017 [64]	ITA	35	17	18	27	68	36	32		27
Lapointe, 2023 [65]	CAN	52			33	69	39			28
Leddy, 2016 [66]	USA	33	21	12	23	57	3	23	22	
Lee, 2008 [67]	USA	48	24	24	28	63	57	33	27	
Linde, 2020 [68]	USA	43			33	56	13		20	30
Macko, 2005 [69]	USA	61	32	29	43	64	37		31	
Munari, 2018 [70]	ITA	15	8	7	14	62	68.4		9	27
Pang, 2005 [71]	CAN	63	32	31	37	65	62.4	37	41	
Reynolds, 2021 [72]	AUS	20	10	10	18	57	3.6	16	10	
Severinsen, 2014 [73]	DEN	29	16	13	31	68	17		16	
Tang, 2009 [74]	CAN	45	23	22	12	65	0.5	17	12	26
Tang, 2014 [75]	CAN	47	22	25	29	66	48	19	31	28

^1^ BMI = Body Mass Index.

**Table 3:** Interventions’ characteristics.

Study	Control			Experimental			Frequency (Sessions/Week)	Program Length (Weeks)	Setting
	Intensity	Type	Duration (min)	Intensity	Type	Duration (min)			
Ahmed, 2021 [51]	“somewhat heavy” on the mRPE	standardized trunk care regime	45	“heavy” in the mRPE	high-intensity multiplanar trunk training coupled with dual-task	45	5	12	outpatients
Boyne, 2016 [52]	45–50% HRR	treadmill	25	determined by a steep ramp test at the end of the warm-up	treadmill	25	3	4	outpatients
Boyne, 2023 [53]	40–60% HRR	overground training and treadmill	45	>60%HRR	overground training and treadmill	45	3	12	outpatients
Gjellesvik, 2020 [54]	moderate-to-high intensity	standard care	not specified	85–95% HRR	treadmill	38	3	8	outpatients
Gjellesvik, 2021 [55]	not specified	standard care	not specified	85–95% HRpeak	treadmill	not specified	3	8	outpatients
Globas, 2012 [56]	not specified	conventional care physiotherapy	60	60–80% HRR	treadmill	30–50	3	18	outpatients
Holleran, 2015 [57]	30–40% HRR	treadmill and overground walking	40	70–80% HRR	treadmill and overground walking	40	3	12	outpatients
Hornby, 2015 [58]	not specified	not specified	60	70–80% HRR or RPE ≥ 14	treadmill and overground walking	60	4–5	10	outpatients
Hornby, 2021 [59]	30–40% HRR	not specified	60	70–80% HRR	treadmill, overground walking, and stair climbing	60	3–5	8	outpatients
Ivey, 2007 [60]	not specified	conventional physical therapy	40	60–70% HRR	treadmill	40	3	24	outpatients
Ivey, 2010 [61]	not specified	conventional physical therapy	40	60–70% HRR	treadmill	40	3	24	outpatients
Ivey, 2015 [62]	50%HRR	treadmill	50	80–85% HRR	treadmill	30	Not specified	24	outpatients
Jin, 2012 [63]	20–30% HRR	low- intensity overground walking training	40	50–70% HRR	aerobic cycling training combined with lower-limb weights	40	5	8	outpatients
Lamberti, 2017 [64]	not specified	endurance phase (weeks 1–4): low-intensity overground walking; mixed phase (weeks 5–8): targeted	30	60–70% HRR	endurance phase (weeks 1–4): treadmill walking; mixed phase (weeks 5–8): gym machines (leg extension and leg curl)	60	3	8	outpatients
Lapointe, 2023 [65]	not specified	walking, swimming, dancing, or cycling; moderate-intensity continuous training (MICT) sessions	20–40	bouts at 95% of peak power output	upright ergocycle; progressive low-volume HIIT sessions and moderate-intensity continuous training (MICT) sessions	20–40	3	24	outpatients
Leddy, 2016 [66]	30–40% HRR	standard physical therapy	60	70–80% HRR or 15–17 RPE	treadmill	60	4	10	outpatients
Lee, 2008 [67]	not specified	sham cycling + sham progressive resistance training (PRT)	30	70% VO2peak	different combinations of real or sham cycling followed by real or sham PRT	30	3	10–12	outpatients
Linder, 2020 [68]	not specified	semi-recumbent stationary bicycles	90	60–80% HRR	semi-recumbent stationary bicycles; forced exercise and repetitive task practice (FE+RTP) or voluntary	90	3	8	outpatients
Macko, 2005 [69]	30–40% HRR	treadmill	40	60–70% HRR	treadmill	40	3	18	outpatients
Munari, 2018 [70]	80% of the self-selected speed and inclination	treadmill	55	85–95% VO2peak	uphill walking training on treadmill device	40	3	12	outpatients
Pang, 2005 [71]	not specified	seated upper-extremity program	60	70–80% HRR	fitness and mobility exercise (FAME) program	60	3	19	outpatients
Reynolds, 2021 [72]	<40% HRR	low-intensity “conventional exercise program”	10 (week 1–4); 20	40–59% HRR	progressive moderate-intensity CV training	30	2	12	outpatients
Severinsen, 2014 [73]	<60% 1RM	low-intensity sham training of the arms	60	75% HRR for aerobic training and 80% of one-repetition maximum (1RM; i.e., the maximal load that can be lifted once) for resistance training	aerobic training (cycle ergometer) and resistance training (training machines)	60	3	12	outpatients
Tang, 2009 [74]	not specified	conventional rehabilitation early after stroke	60	50–75% higher peak work rate (WR)	aerobic cycle ergometer training + conventional rehabilitation early after stroke	30 cycle ergometer + 60 conventional physical therapy	3	5	inpatients
Tang, 2014 [75]	<40% HRR	low-intensity balance and flexibility (BF) program	30–40	70–80% HRR	high-intensity aerobic exercise (AE) program	60	3	24	outpatients

**Table 4:** Outcome measures.

Author	Year	Pre-treatment		Post-Treatment		*p*-Value		
		Experimental	Control	Experimental	Control	Exp vs. Cont	Within Exp	Within Cont
6 MWT								
Boyne [52]	2016	220	247	235	262			
Boyne [53]	2023	196	177	4 wk = 223 8 wk = 254 12 wk = 267	4 wk = 189 8 wk = 206 12 wk = 204	0.44 4 wk = 0.28 8 wk = 0.02 12 wk = 0.005		
Gjellesvik [55]	2021	480.30	550.70	520.00	542.60	0.03		
Globas [56]	2012	274.4 ± 113	332.1 ± 138	261.2 ± 177	265.9 ± 189	<0.001		
Holleran [57]	2015	191 ± 93	207 ± 123	231 ± 121	213 ± 125	<0.01		
Hornby [58]	2016	116 ± 88	131 ± 108	232 ± 149	160 ± 111	0.001		
Hornby [59]	2019	HV 212 HF 197	197	HV 82 HF 96	34	0.96		
Ivey [62]	2015	780 ± 105	564 ± 73	964 ± 131	668 ± 76	0.22	<0.001	0.06
Jin [63]	2012	212.0 ± 63.5	212.2 ± 50.1	218.5 ± 63.7	213.5 ± 50.6	<0.001	<0.001	<0.001
Lamberti [64]	2017	258 ± 133	230 ± 107	292 ± 136	301 ± 132	0.009	<0.01	<0.01
Lee [67]	2008	comb = 266.0 ± 123.5 AT = 249.3 ± 158.3 PRT = 239.8 ± 141.0	273.2 ± 162.1	comb = 290.2 ± 136.2 AT = 261.5 ± 162.7 PRT = 247.2 ± 148.8	278.1 ± 162.1	0.06	0.11	0.31
Linder [68]	2020	F 417 V 295	335	F 480 V 327	312	pre 0.02 post 0.07		
Macko [69]	2005	761±73	848 ± 109	922 ± 79	868 ± 100	0.018	<0.001	
Munari [70]	2018	316.62 ± 115.34	294.24 ± 122.95	380.87 ± 121.36	300.34 ± 114.91	0.005	0.012	0.236
Pang [71]	2005	328.1	304.1	392.7	342.4	0.025		
Reynolds [72]	2021	400.9	344.8	443.4	370.9	0.59	0.11	0.18
Severinsen [73]	2014	AT = 313 PRT = 287	307	AT = 332 PRT = 316.6	350.5	0.0911		
Tang [74]	2009	207.0 ± 46.6	198.9 ± 40.2	334.2 ± 33.1	288.4 ± 38.9	0.23	<0.001	<0.001
Tang [75]	2014	278.2 ± 128.5	322.2 ± 142.4	298.1 ± 134.2	331.5 ± 149.2	0.39		
VO2peak								
Boyne [52]	2016	16	21.6	18.2	20.3	0.02		
Boyne [53]	2023	11.5	10.9	4 wk = 12.8 8 wk = 13.5 12 wk = 13.4	4 wk = 11.3 8 wk = 12.6 12 wk = 12.3	0.55 4 wk = 0.16 8 wk = 0.72 12 wk = 0.60		
Gjellesvik [54]	2020	31.83	33.35	34.88	31.76	0.001		
Globas [56]	2012	18.9 ± 4.6	21.7 ± 7.8	24.4 ± 6.6	20.9 ± 7.8	<0.001		
Holleran [57]	2015	11 ± 5.5	9.5 ± 3.7	11 ± 3.6	11 ± 4.8	0.48		
Ivey [60]	2007	13.7 ± 0.9	14.8 ± 0.9	15.7 ± 1.1	14.4 ± 1.0	0.002	<0.05	
Ivey [61]	2010	14.1 ± 4.0	13.5 ± 3.6			pre = 0.54		
Ivey [62]	2015	15.9 ± 1.7	21.3 ± 1.6	16.6 ± 1.1	17.5 ± 1.2			
Jin [63]	2012	13.2 ± 0.9	13.2 ± 1	16.8 ± 1	13.3 ± 1	<0.001	<0.001	
Lapointe [65]	2023	H + MC: 18.9 ± 5.5 MC: 21.1 ± 4.5	19.3 ± 8.4	H + MC: 21.9 ± 6.1 MC: 24.1 ± 4.9	18.7 ± 8.0	0.297	<0.001	
Leddy [66]	2016	15 ± 5.2	14 ± 3.6	21 ± 9.1	15 ± 3.6	<0.01		
Lee [67]	2008	comb = 14.4 ± 3.1 AT = 13.0 ± 4.5 PRT = 14.0 ± 3.3	13.5 ± 3.5	comb = 16.6 ± 5.2 AT = 14.5 ± 3.9 PRT = 13.5 ± 3.8	12.7 ± 4.3	0.03	0.002	0.51
Macko [69]	2005	14.9 ± 0.9	14.7 ± 1	17.3 ± 1	14.9 ± 1	0.018	<0.001	
Munari [70]	2018	20.88 ± 5.28	20.49 ± 5.58	25.48 ± 4.03	19.63 ± 2.87	0.015	0.025	0.753
Pang [71]	2005	22.5	21.5	24.5	21.8	0.034		
Reynolds [72]	2021	17.5	14.4	20.1	17.7	0.75	0.02	0.01
Severinsen [73]	2014	AT = 18 PRT = 16	15	AT = 20.5 PRT = 16.6	15.06	0.0015		
Tang [74]	2009	11.6 ± 0.7	11.2 ± 0.5	13.1 ± 0.9	12.1 ± 0.8	0.71	0.004	0.004
Tang [75]	2014	16.9 ± 7.1	16.9 ± 6.1	17.4 ± 7.0	16.6 ± 5.3	0.85		
10 MWT								
Ahmed [51]	2021	0.44 ± 0.14	0.47 ± 0.16	0.79 ± 0.17	0.64 ± 0.16	0.32	<0.001	<0.001
Boyne [52]	2016	0.63	0.76	0.73	0.78	0.62		
Boyne [53]	2023	S 0.52 F 0.70	S 0.49 F 0.62	S 4 wk = 0.63 8 wk = 0.66 12 wk = 0.71 F 4 wk = 0.92 8 wk = 0.94 12 wk = 0.98	S 4 wk = 0.51 8 wk = 0.55 12 wk = 0.55 F 4 wk = 0.63 8 wk = 0.71 12 wk = 0.71	S 0.56 4 wk = 0.009 8 wk = 0.04 12 wk = 0.003 F 0.33 4 wk = <0.001 8 wk = 0.003 12 wk = 0.002		
Gjellesvik [55]	2021	1.75	1.96	1.79	1.88	0.624		
Globas [56]	2012	C = 0.73 ± 0.28 M = 0.91 ± 0.34	C = 0.70 ± 0.44 M = 0.88 ± 0.56	C = 0.79 ± 0.29 M = 1.02 ± 0.38	C = 0.70 ± 0.46 M = 0.87 ± 0.62	<0.001		
Ivey [62]	2015	S = 21.3 ± 3.4 F = 15.4 ± 2.1	S = 24.0 ± 2.9 F = 17.2 ± 2.2	S = 20.9 ± 4.3 F = 13.8 ± 2.1	S = 20.7 ± 2.6 F = 15.9 ± 1.9	S = 0.13 F = 0.81	S = 0.76 F = 0.001	S = 0.03 F = 0.34
Lamberti [64]	2017	1.03 ± 0.46	0.98 ± 0.41	1.21 ± 0.53	1.18 ± 0.47			<0.01
Macko [69]	2005	UP 0.63 ± 0.06 FP 0.82 ± 0.08	UP 0.67 ± 0.07 FP 0.9 ± 0.10	UP 0.74 ± 0.06 FP 0.95 ± 0.09	UP 0.76 ± 0.08 FP 1 ± 0.11	UP 0.707 FP 0.970	UP < 0.01 FP < 0.01	UP <0.01 FP <0.01
Munari [70]	2018	11.81 ± 3.83	12.24 ± 4.09	10.05 ± 2.53	12.88 ± 4.43	0.007	0.042	0.102
Reynolds [72]	2021	UP 1.16 FP1.47	UP 0.95 FP 1.2	UP 1.2 FP 1.57	UP 0.97 FP 1.31	UP 0.82 FP 0.96	UP 0.53 FP 0.35	UP 0.74 FP 0.13
Severinsen [73]	2014	AT = 0.81 RT = 0.87	0.89	AT = 0.86 RT = 0.96	1.01	0.0037		
BBS								
Ahmed [51]	2021	35.83 ± 7.10	35.93 ± 7.46	45.14 ± 6.58	41.36 ± 7.82	0.95	<0.001	
Gjellesvik [55]	2021	53	54.3	53.6	52.9	0.025		
Globas [56]	2012	49.3 ± 6.5	45.2 ± 11.0	51.1 ± 6.4	44.3 ± 11.9	<0.05		
Hornby [58]	2016	32 ± 16	33 ± 16	40 ± 11	38 ± 14	0.660		
Jin [63]	2012	47.9 ± 3.1	47.4 ± 3.7	48.6 ± 2.9	48.3 ± 3.9	0.228	<0.01	
Lamberti [64]	2017	50 ± 5	49 ± 5	52 ± 4	53 ± 3		<0.01	
TUG								
Ahmed [51]	2021	22.74 ± 5.20	23.48 ± 4.49	12.12 ± 3.60	15.86 ± 5.46	0.49	<0.001	<0.001
Gjellesvik [55]	2021	9.24	8.25	9.02	8.7	0.771		
Munari [70]	2018	13.14 ± 3.83	12.94 ± 5.04	11.70 ± 3.51	12.03 ± 3.87	0.810	0.119	0.257

HV: high-intensity stepping training in variable contexts (high variable [HV]). HF: high-intensity stepping training forward on a treadmill and overground with minimal variability (high forward [HF]); AT: aerobic training, PRT: progressive resistance training, comb: combined aerobic and resistance; F: forced aerobic exercise; V: voluntary aerobic exercise; MC: moderate-intensity continuous training; H: high-intensity interval training; S: self-selected gait speed, F: fastest gait speed; C: comfortable walking speed, M: maximum walking speed; UP: usual pace; FP: fast pace.

## Data Availability

The data presented in this study are available within the text and in the Supplementary Materials.

## Supplementary Materials

The following supporting information can be downloaded at: https://www.mdpi.com/article/10.3390/jcm13185498/s1, Figure S1: Search Strategy for PubMed; Figure S2: Search strategy for Cochrane, EBSCO, EMBASE, and Scopus, Table S1: Risk of bias assessment, Table S2: Sensitivity analysis.
