# Supplementary material for: High-Intensity Exercise Training Impact on Cardiorespiratory Fitness, Gait Ability, and Balance in Stroke Survivors: A Systematic Review and Meta-Analysis
Source: J Clin Med. 2024 Sep 17;13(18):5498. doi: 10.3390/jcm13185498 (PMC11432212; doi:10.3390/jcm13185498)
Supplement: Supplementary file 1 [file jcm-13-05498-s001.zip › jcm-3142503 Table S1.pdf]

Table S1. Risk of bias assessment.

	Bias arising from the randomisation process (D1)				Bias due to deviations from intended interventions (D2)								Bias due to missing outcome data (D3)					Bias in measurement of the outcome (D4)						Bias in selection of the reported result (D5)				Overall judgment
First author, year	1,1	1,2	1,3	Risk-of-bias judgment	2,1	2,2	2,3	2,4	2,5	2,6	2,7	Risk-of-bias judgment	3,1	3,2	3,3	3,4	Risk-of-bias judgment	4,1	4,2	4,3	4,4	4,5	Risk-of-bias judgment	5,1	5,2	5,3	Risk-of-bias judgment	
Ahmed, 2021	y	y	n	low	py	py	pn	na	na	y	na	some concerns	y	na	na	na	low	n	pn	py	pn	pn	low	y	pn	pn	low	low
Munari, 2016	y	y	n	low	py	py	pn	na	na	y	na	some concerns	y	na	na	na	low	n	n	py	n	n	low	y	n	n	low	low
Boyne, 2016	y	y	n	low	y	n	ni	pn	na	y	na	some concerns	y	na	na	na	low	n	py	ni	pn	na	low	y	pn	pn	low	low
Boyne, 2023	y	y	n	low	n	n	na	na	na	y	na	low	y	na	na	na	low	n	pn	ni	na	na	low	y	n	n	low	low
Pang, 2005	y	y	n	low	y	n	pn	na	na	y	na	some concerns	y	na	na	na	low	n	n	ni	n	n	low	y	n	n	low	low
Reynolds, 2021	y	y	n	low	n	pn	na	na	na	y	na	low	y	na	na	na	low	n	n	n	na	na	low	y	n	n	low	low
Lamberti,2017	y	y	n	low	ni	y	py	ni	na	y	na	some concerns	y	na	na	na	low	n	n	n	na	na	low	y	n	n	low	low
Lapointe, 2023	y	y	n	low	py	n	pn	na	na	y	na	some concerns	y	na	na	na	low	n	n	n	na	na	low	y	n	n	low	low
Gjellesvik, 2020	y	pn	n	low	py	pn	pn	na	na	y	na	some concerns	y	na	na	na	low	n	pn	n	na	na	low	y	n	n	low	low
Gjellesvik, 2021	y	y	n	low	n	y	pn	na	na	y	na	low	pn	pn	py	ni	high	n	n	py	py	pn	some concerns	y	n	n	low	some concerns
Leddy, 2016	y	y	n	low	py	pn	pn	na	na	y	na	some concerns	y	na	na	na	low	n	py	n	na	na	low	y	n	n	low	low
Globas 2012	y	y	n	low	y	py	pn	na	na	y	na	some concerns	y	na	na	na	low	n	py	ni	py	py	some concerns	y	n	n	low	some concerns
Lee, 2008	y	y	n	low	n	n	na	na	na	y	na	low	py	na	na	na	low	n	n	y	pn	na	low	y	n	n	low	low

Linder, 2020	y	y	n	low	pn	n	na	na	na	y	na	low	py	na	na	na	low	n	n	py	pn	na	low	y	n	n	low	low
Holleran, 2015	y	y	n	low	py	y	pn	na	na	y	na	some concerns	y	na	na	na	low	n	pn	y	pn	na	low	y	n	n	low	low
Severinsen, 2014	y	py	n	low	pn	py	n	na	na	y	na	some concerns	y	na	na	na	low	n	n	n	na	na	low	y	n	n	low	some concerns
Tang, 2009	y	n	n	high	ni	ni	n	na	na	y	na	some concerns	y	na	na	na	low	n	n	ni	n	na	low	y	n	n	low	some concerns
Tang, 2014	y	y	n	low	ni	n	n	na	na	y	na	low	y	na	na	na	low		n	n	na	na	low	y	n	n	low	low
Hornby, 2021	y	y	n	low	py	y	pn	na	na	y	na	some concerns	y	na	na	na	low	n	pn	y	pn	na	low	y	n	n	low	low
Hornby, 2015	y	y	n	low	n	n	na	na	na	y	na	low	y	na	na	na	low	n	pn	ni	na	na	low	y	n	n	low	low
Ivey, 2015	y	y	n	low	py	py	pn	na	na	y	na	some concerns	y	na	na	na	low	n	n	py	n	n	low	y	n	n	low	low
Ivey, 2007	y	y	n	low	py	py	pn	na	na	y	na	some concerns	y	na	na	na	low	n	pn	py	pn	pn	low	y	pn	pn	low	low
Ivey, 2010	y	y	n	low	y	n	ni	pn	na	y	na	some concerns	y	na	na	na	low	n	py	ni	pn	na	low	y	pn	pn	low	low
Jin, 2012	y	y	n	low	py	y	pn	na	na	y	na	some concerns	y	na	na	na	low	n	pn	y	pn	na	low	y	n	n	low	low
Macko, 2005	y	y	n	low	y	py	na	na	na	y	na	some concerns	y	na	na	na	low	n	pn	py	pn	n		y	n	n	low	low
