# Supplementary material for: High-Intensity Exercise Training Impact on Cardiorespiratory Fitness, Gait Ability, and Balance in Stroke Survivors: A Systematic Review and Meta-Analysis
Source: J Clin Med. 2024 Sep 17;13(18):5498. doi: 10.3390/jcm13185498 (PMC11432212; doi:10.3390/jcm13185498)
Supplement: Supplementary file 1 [file jcm-13-05498-s001.zip › Supplementary Figure S1 and S2_ Search Strategies (1).pdf]

Supplementary Figure S1: Search Strategy for PubMed

Stroke[Mesh] OR "cerebrovascular accident"[tw] OR "cerebrovascular disorder"[tw] OR "ischemic stroke"[tw] OR “hemorrhagic stroke”[tw] OR CVA [tw] OR CVAs [tw] OR “cerebrovascular apoplexy”[tw] OR “apoplexy”[tw] OR "intracerebral hemorrhage" [tw] OR "cerebral hemorrhage" [tw] OR "cerebral infarction"[tw] OR "brain infarction"[tw] OR "brain ischemia"[tw] OR ((“stroke” [Mesh] OR “stroke” [tw]) AND (“cerebrum” [Mesh] OR “cerebrum” [tw] OR “cerebral” [tw] OR “brain” [Mesh] OR “brain” [tw]))

AND

(
"intensity training"[tw] OR "intensity exercise"[tw] OR "aerobic intensity"[tw] OR (intens*[tw] AND (exercise [mesh] OR training [tw] OR "physical activit*"[tw] OR “physical therapy”[tw] OR "physical education and training"[Mesh] OR sports[mesh] OR "exercise therapy"[tw] OR "motor activity"[Mesh])) OR “aerobic interval training”[tw] OR (“aerobic interval”"[tw] AND (exercise [Mesh] OR “training”[tw] OR “physical activit*”[tw] OR “physical therapy”[tw] OR “physical education and training”[Mesh] OR sports [Mesh] OR “exercise therapy”[tw] OR “motor activity”[Mesh]))
OR
"high intensity training"[tw] OR "high intensity exercise"[tw] OR "high intensity"[tw] OR "high-aerobic intensity" OR (“high intens*”[tw] AND (exercise [mesh] OR training [tw] OR "physical activit*"[tw] OR “physical therapy”[tw] OR "physical education and training"[Mesh] OR sports[mesh] OR "exercise therapy"[tw] OR "motor activity"[Mesh])) OR "continuous moderate exercise"[tw] OR ("continuous"[tw] AND "moderate"[tw] AND (exercise [mesh] OR training [tw] OR "physical activit*"[tw] OR “physical therapy”[tw] OR "physical education and training"[Mesh] OR sports[Mesh] OR "exercise therapy"[tw] OR "motor activity"[Mesh]))
)

Supplementary Figure S2: Search strategy for Cochrane, EBSCO, EMBASE and Scopus

Stroke OR cerebrovascular accident OR cerebrovascular disorder OR ischemic stroke OR hemorrhagic stroke OR CVA OR CVAs OR cerebrovascular apoplexy OR apoplexy OR intracerebral hemorrhage OR cerebral hemorrhage OR cerebral infarction OR brain infarction OR brain ischemia

AND

(
intensity training OR intensity exercise OR aerobic intensity OR (intensity AND exercise) OR training OR physical activity OR physical therapy OR physical education and training OR sports OR exercise therapy OR motor activity OR aerobic interval training OR (aerobic interval AND exercise OR training OR physical activity OR physical therapy OR physical education and training OR sport OR exercise therapy OR motor activity)
OR
high intensity training OR high intensity exercise OR high intensity OR high-aerobic intensity OR (high intensity AND (exercise OR training OR physical activity OR physical therapy OR physical education and training OR sports OR exercise therapy OR motor activity)) OR continuous moderate exercise OR (continuous AND moderate AND (exercise OR training OR physical activity OR physical therapy OR physical education and training OR sports OR exercise therapy OR motor activity))
)
