# Supplementary material for: High-Intensity Exercise Training Impact on Cardiorespiratory Fitness, Gait Ability, and Balance in Stroke Survivors: A Systematic Review and Meta-Analysis
Source: J Clin Med. 2024 Sep 17;13(18):5498. doi: 10.3390/jcm13185498 (PMC11432212; doi:10.3390/jcm13185498)
Supplement: Supplementary file 1 [file jcm-13-05498-s001.zip › Supplementary Table S2_ Sensitivity analysis .pdf]

Table S1. Sensitivity analysis.

	N	pMD	95%CI	p-value heterogeneity	I^2
**Sensitivity analysis**					
Intervention arm with greater efficacy	19	97.9	[47.22; 148.58]	< 0.0001	96.40%
Term follow-up main analysis	6	95.2469	[24.0335; 166.4602]	< 0.0001	90.80%
Term follow-up most effective intervention	6	32.6371	[-2.4659; 67.7402]	0.0657	54.70%
**Treatment duration strata**					
<12	10	81.63	[25.82; 137.44]	0.3047*	94.60%
12	4	31.32	[-22.75; 85.40]		75.90%
>12	5	139.13	[-43.54; 321.81]		98.60%

a) **6MWT sensitivity analysis**

*p-value test layer comparison

	N	pMD	95%CI	p-value heterogeneity	I^2
**Sensitivity analysis**					
Intervention arm with greater efficacy	19	4.0876	[2.7666; 5.4086]	< 0.0001	97.30%
Term follow-up main analysis	5	5.626	[-1.6256; 12.8776]	< 0.0001	97.10%
Term follow-up most effective intervention	5	5.2689	[-0.8767; 11.4146]	< 0.0001	96.40%
**Treatment duration strata**					
<12	6	4.421	[1.6071; 7.2350]	0.7086*	98.70%
12	4	2.6545	[-0.4267; 5.7357]		82.30%
>12	8	3.594	[2.0307; 5.1573]		94.00%

b) **VO2peak sensitivity analysis**

*p-value test layer comparison

	N	pMD	95%CI	p-value heterogeneity	I^2
**Sensitivity analysis**					
Intervention arm with greater efficacy	11	0.075	[-0.0893; 0.2382]	< 0.0001	95.00%
Term follow-up main analysis	5	0.2901	[0.0332; 0.5471]	< 0.0001	95.20%
Term follow-up most effective intervention	5	0.2988	[-0.0362; 0.6339]	< 0.0001	94.70%
**Treatment duration strata**					
<12	3	0.077	[-0.0018; 0.1550]	0.9263*	8.80%
12	3	0.121	[-0.1770; 0.4185]		96.50%
>12	5	0.091	[0.0584; 0.1235]		0.00%

c) **10MWT sensitivity analysis**

*p-value test layer comparison
